# Co‐Creating Preconception Health Content for a Digital Resource for Women From Diverse Ethnic Backgrounds

**DOI:** 10.1111/hex.70542

**Published:** 2026-04-09

**Authors:** Asvini K. Subasinghe, Sanduni Madawala, Dinali Fernando, Jue Xie, Jessica Watterson, Ling Wu, Chris Prawira, Patrick Olivier, Jacqueline A. Boyle

**Affiliations:** ^1^ Department of Health Systems and Equity, Eastern Health Clinical School Monash University Melbourne Australia; ^2^ Action Lab, Faculty of Information Technology Monash University Melbourne Australia

## Abstract

**Introduction:**

Planning pregnancy and optimal health prior to pregnancy can significantly reduce pregnancy complications and poor maternal health outcomes. Women from culturally and racially marginalised groups experience more unintended pregnancies, increased preconception risk factors and adverse pregnancy outcomes, including maternal and infant mortality, compared to native born Australians. Improving health literacy through culturally relevant and accessible resources may improve preconception health. We have previously shown that women from migrant and refugee backgrounds would prefer receiving preconception information on digital platforms. The aim of this study is to understand the focus and concerns of the women with regard to the content for a digital resource called ‘BabyReady?’.

**Methods:**

We conducted virtual workshops with 10 women from East Asian, South Asian, Central Asian, Middle‐Eastern, and African backgrounds to understand more about cultural practices related to pregnancy preparation, preconception information sources and to identify topics that may be useful to include on a digital dashboard. Focus group discussions were transcribed and a content analysis was conducted.

**Results:**

A range of culturally specific practices included eating warm foods, using acupuncture and alternative medicines to prepare for pregnancy. Expectations from family and friends induced high levels of stress and feeling controlled. Relatives were integral in decision making about pregnancy planning, particularly the mother‐in‐law. Women wanted to learn more about egg freezing, government benefits, adverse birth outcomes, how to navigate the healthcare system, interpreter services and where they could locate female health professionals.

**Conclusion:**

Digital preconception health content may be optimised for use by women from ethnically diverse backgrounds if it includes information around stress management, how to balance cultural expectations and beliefs with health advice, how to locate female health practitioners who speak their language and appropriate pregnancy planning.

**Patient or Public Contribution:**

The public participated in workshops providing feedback on what digital culturally responsive preconception health content means to them and how best to integrate it into a digital health dashboard.

## 1 Introduction

Australia's National Women's Health Strategy priorities include increased access to information, and improved health promotion for sexual and reproductive and preconception health [1]. It also recognises priority groups with unmet need including women from migrant and refugee backgrounds and those living in rural areas [1]. These women face difficulties receiving advice on pregnancy planning and optimal preconception health, due to cultural differences, geographical limitations, language difficulties and cost which leads to poorer pregnancy outcomes and unintended pregnancy [2, 3].

Women from migrant and refugee backgrounds have greater rates of adverse pregnancy outcomes, including gestational diabetes, high blood pressure in pregnancy and maternal and infant mortality, compared to native born Australians [4, 5, 6]. Women from East Asian, South Asian, Central Asian, Middle‐Eastern, and African backgrounds are particularly at high risk of suboptimal pregnancy outcomes compared to women from English‐speaking backgrounds [6, 7]. A lack of knowledge, health literacy and access to services are some of the factors related to adverse pregnancy outcomes in these women [8].

Despite the availability of globally designed preconception care tools, these have not been designed for use among culturally diverse groups [9]. This means that a large group of women with lower rates of health literacy and those at greatest risk of poor maternal and foetal outcomes cannot access important information and tools that can improve pregnancy outcomes [8]. There are strong data to support tools that are co‐designed with women, rather than for them, are better positioned to meet cultural, linguistic, and contextual needs [10, 11, 12]. We have previously reported that women from migrant and refugee backgrounds would prefer receiving preconception information as digital resources [13, 14, 15]. However, women noted they prefer a digital resource that is easy to navigate, and customised to meet their needs and preferences [14]. *Delivering personalised preconception health information through a digital platform aims to empower these women to seek appropriate support for a safe and successful pregnancy* [15].

*BabyReady?* is designed to provide tailored advice and support to women about pregnancy planning depending on their intention to currently prepare for, or prevent pregnancy. *BabyReady?* builds on our work co‐designing an adaptation of a health education and behaviour change technology ‘Gabby’ with colleagues in Boston to meet the identified needs of Australian women [14]. Extending beyond medical information, *BabyReady?* will also provide tailored information and leverage other women's lived experiences to address three additional pillars: Social and emotional wellbeing, relationships and social connections, and resources (e.g., workplace and financial readiness). The personalised information will utilise multiple forms of delivering health promotion content to engage women from these priority groups, such as podcasts, downloadable information sheets, and videos, to improve health literacy and pregnancy outcomes and reduce the risk of unintended pregnancy. Conversational and generative artificial intelligence (AI) technologies will be utilised to curate media recommendations and advice tailored to user needs and preferences.

Therefore, the aim of this study was to co‐develop culturally responsive digital preconception health content for women from East Asian, South Asian, Central Asian, Middle‐Eastern, and African backgrounds to be embedded within BabyReady?

## 2 Methods

Ethics approval was sought from the Monash University Human and Research Ethics Committee (Project ID: 40436). Participants were provided with a participant information sheet and consent was implicit upon attending workshops.

### 2.1 Phase 1

We conducted a 2‐h audio recorded virtual workshop for women from East Asian, South Asian, Central Asian, Middle‐Eastern, and African backgrounds. Participants were compensated for their time with a $40 gift voucher for every hour of their input. Consumers viewed a presentation on the aims and objectives of our study. We then formed two breakout rooms to explore the following questions:

- What does culturally responsive content mean to you?
- What does a customised digital health platform look like to you?

We invited participants back into the main room and recorded responses from each group. In the last part of the workshop, we formed four breakout rooms to facilitate discussion about lived experiences across four themes, previously identified and integral to optimising preconception health: Mental and physical health, social connections, sleep and diet [14]. We invited participants back into the main room and recorded suggestions around culturally responsive content for each theme.

### 2.2 Phase 2

Qualitative analysis: Focus group discussions were transcribed and thematic analysis was conducted using NVivO software by S.M. and cross‐checked by A.K.S. Transcripts were first coded inductively using Braun and Clarke's reflexive thematic analysis. Data were read and re‐read to become familiar with the content, generate initial data‐driven codes, and then iteratively organise these into broader themes following inductive coding and theme development, codes/themes were mapped to Capability, Opportunity, Motivation‐Behaviour domains (by two researchers, with discrepancies resolved through discussion).

### 2.3 Phase 3

Consumers were invited back to participate in a 2‐h online workshop where they were presented with a beta version of the digital dashboard with preconception health content specifically curated by our team and understand whether we integrated their feedback appropriately.

## 3 Results

We reached data saturation when gathering feedback from 10 participants from East Asian, South Asian, Central Asian, Middle‐Eastern, and African backgrounds (Table 1). All women were aged between 25 and 45 years of age and represented high cultural and linguistic diversity as described in the table.

**Table 1:** Participant characteristics.

	N (%)
Ethnic background	
East Asian	5 (50)
South Asian	2 (20)
Central Asian	1 (10)
Middle Eastern	1 (10)
African	1 (10)
Migration status	
**< **10 years	4 (40)
> 10 years	6 (60)
Language fluency	
English	10 (100)
Chinese	5 (50)
Arabic	1 (10)
Darsi	1 (10)
Urdu	1 (10)
Sinhalese	1 (10)
Education	
Tertiary educated	10 (100)

Three major themes were identified from the workshop after following the COM‐B theoretical framework (Table 2): Culturally specific practices, main sources of preconception information and health promotion strategies for digital resources for women from diverse backgrounds.

**Table 2:** Mapping of themes and exemplar quotes to COM‐B domains and design implications.

COM‐B domain	Sub‐component	Theme	Sub‐theme/code	Exemplar quote	Design implications for the BabyReady resource
Capability	Psychological (health literacy)	Culturally specific practices	Understanding ‘preconception care’ terminology	‘I don't even think English speakers know what that means’	Start the resource with a plain‐language definition of ‘preconception care’ + visual explainer; glossary and hover tooltips.
Capability	Psychological (myth‐busting)	Culturally specific practices	‘Eating for two’/diet traditions		Short myth‐busting cards with evidence checks; culturally sensitive nutrition tips; downloadable conversation guide for family.
Capability	Psychological (knowledge)	Best strategies for digital	Egg‐freezing info is daunting/complex		Balanced, non‐directive primer on fertility options (incl. eligibility, risks, costs, wait times) with links to reputable services.
Capability	Psychological (practical literacy)	Best strategies for digital	Government benefits and costs (Centrelink)		‘Money and benefits’ pathway: Step‐by‐step eligibility, timelines, and links; printable checklist.
Opportunity	Social (norms/influence)	Culturally specific practices	Family pressure/over‐feeding; role of relatives	‘what role don't they (relatives) play?…Alot…’	Modules on negotiating expectations; scripts for boundary‐setting with family; partner/relative pages summarising ‘how to support’.
Opportunity	Social (power dynamics)	Best strategies for digital	Husband as default translator/‘middle‐man’		Full multilingual UX; encourage use of female interpreters; interpreter‐booking how‐to; privacy prompts before sensitive topics.
Opportunity	Social (safety)	Culturally specific practices	Perceived rise in family violence	‘treated like an object… very detrimental’	Discreet safety exit button; inline prompts on recognising coercion; quick links to 24/7 helplines; private‐mode viewing tips.
Opportunity	Physical/Environmental (language)	Best strategies for digital	Language‐concordant care and content	‘someone from their cultural background get straight to the point’	Language toggles; audio narration; dialect notes; imagery that reflects communities; female cultural worker ‘voices’ in content.
Opportunity	Physical/Environmental (navigation)	Best strategies for digital	Health‐system navigation (GP as gatekeeper)		‘How the system works’ flowchart; referral pathway explainer; pre‐GP question prompts; map of services by postcode.
Opportunity	Physical/Environmental (accessibility)	Best strategies for digital	Reduce wordiness; more translation; audio and icons		Plain‐language rewrite; audio for all pages; icon‐first navigation; offline PDFs; low‐data mode.
Opportunity	Personalisation	Best strategies for digital	Identity nuance (e.g., Chinese‐Australian but can't read Chinese)		Onboarding that asks preferred language for reading/listening; per‐user language/format defaults; content picker by topic, not ethnicity labels.
Motivation	Reflective (values/intentions)	Culturally specific practices	Career/age pressure; ‘beneficial’ care vs. cultural practice		Values‐clarification exercises; planning worksheets balancing career/family; ‘what matters to me’ goal cards.
Motivation	Reflective (role‐modelling)	Best strategies for digital	Lived experience stories and podcasts		Podcast series (30–60 min conversational tone) with diverse women; topic tags (mental health, cultural pressures, shared parenting).
Motivation	Reflective (social roles)	Best strategies for digital	Involving men/fatherhood		Dedicated partner track: ‘How to support before pregnancy’; co‐designed episode on male roles and shared responsibilities.
Motivation	Automatic (emotions/habit)	Culturally specific practices	Stress, stigma around mental health help‐seeking	‘labelled as a crazy’	Normalising messages; quick self‐check tools; warm handovers to local services; anonymised Q&A.
Motivation	Automatic (engagement cues)	Best strategies for digital	Topic resonance varies across cultures		

### 3.1 Theme 1: Culturally Specific Practices

Participants discussed culturally specific practices that either hindered them from receiving ‘beneficial’ pre‐conception care, practices they perceived to be of benefit and practices that may not have had a significant benefit physiologically, but were interpreted as providing comfort, care and support to the participants.

Specific dietary practices were described by participants, with family members wanting to over‐feed emphasised as an unhealthy habit specifically experienced by women during their pregnancy. ‘Pressure’ from own self, family and friends were seen as detrimental to the health of the expectant mother. Age of the woman prior to pregnancy and discussions around career also added to the stress for women of reproductive age. Family violence was perceived to increase around the period of preconception, conception and during pregnancy.

‘treated like an object that needs to be protected more. In some cases, there is limits but in other cases … it is very detrimental.’

It was specifically identified that most cultures did not have a specific word for ‘preconception care’. However, most participants thoughts preconception care was conceptually related to preparing the body and ensuring balance is maintained.

‘I don't even think English speakers know what that means. It's quite complicated, unless you're studying it, unless you're a researcher…’

### 3.2 Theme 2: Main Sources of Preconception Information

Participants reported important sources for receiving preconception care information. Most participants identified their general practitioner (GP) or doctor as an important source of information. Parents and in‐laws, relatives' parents, parents and grandparents, friends from similar cultural background particularly those with children were also cited as a main source of information. Social media groups such as Facebook were important.

A digital platform specific to the East Asian community worldwide was identified, ‘Little Red Book (Xiaohongshu)’ and described as a similar platform to Instagram where you are able to interact and comment. Websites discussing the topic of ‘raising kids’ were also identified.

The role of relatives in preconception care and care for the mother during pregnancy was also described. The majority of participants reported the influence of relatives can lead to unnecessary pressure and mental health issues. Decision making was also influenced by relatives where marriage was often perceived by relatives as leading to having babies and these ideas were forceful and sources of stress, described as being ‘down your throat’. Sources of stress for the women included the mother‐in‐law, being ‘labelled as a crazy’ (stigma) if wanting to seek psychological help and complicated family issues.

‘what role don't they (relatives) play?…Alot…’

### 3.3 Theme 3: Health Promotion Strategies for Digital Resources for Women From Diverse Backgrounds

Participants discussed their information needs as required from the digital resource and best health promotion strategies for digital resources for migrant and refugee backgrounds.

Participants discussed that a health practitioner who speaks in their language can help avoid the ‘middle‐man’ translator. This is particularly important when the middle man is a male family member, often the husband, who was perceived by participants to receive more information and have better English. This created an unequal power dynamic meaning the woman had less decision‐making power regarding their preconception, conception and pregnancy. A strong need for female interpreters and female cultural workers was identified. Participants tended to agree the digital resource would have limited effect if it were to only be in English.

‘…whereas if this was someone from their cultural background, they'll skip that … question and get straight to the point of “okay so what is it that you actually need”’

Navigating the Australian healthcare system was described as ‘complicated’ where participants perceived the system to differ greatly from other countries including other westernised nations. The GP was described as the ‘gatekeeper’ for women accessing important information. Fertility was discussed and used as an example to explain this by consumers representing the Middle Eastern community. There were concerns that the GP may not provide specific information unless they had specialised qualifications in women's health. Participants described the need for their GP to ask and enquire about plans regarding pregnancy, family planning and contraception use. This was highlighted as particularly important for people who are part of communities that are known for having ‘back to back pregnancies’ which was highlighted by participants as detrimental to the women's health. Participants described that women had difficulty discussing and explaining issues related to pre‐conception, planning and conception with their GP. This extended to digital platforms where women indicated they are more likely to prefer a platform in their native language regardless of education level.

Other factors that were considered to be important by women were: Egg‐freezing, myth busting old wives' tales so that information could be checked for validity, and resources around financial support and government benefits. Additionally, a resource that was culturally appropriate using respectful language was cited to be important to participants. Identifying someone as ‘Middle Eastern’ or ‘Arab’ was stated to be confusing considering the diversity of groups. The main focus should be on ensuring information is accessible rather than focusing solely on terminology or with which ethnicity people identify. However, asking people who they identify as in an eligibility form was important to tailor the resource and information. It was important to track nuances in identity, for example someone may identify as Chinese‐Australian but may not be able to read the Chinese version of the digital resource.

Finally, all women reported that they would like to hear more lived experiences from other women who have had children and experienced difficult situations or cultural pressures during their conception period. Women suggested that having podcasts available on the platform would be useful to users in listening to real life experiences from people with whom they can relate.

### 3.4 Feedback on Beta Version of BabyReady?

Feedback on integrating a podcast section into the app emphasised the desired tone and format of the resource with a consensus that 30 to 60‐min conversational style episodes would be most accessible for mothers. Key topics women felt were relevant for podcast episodes included cultural impacts on parenthood, the importance of mental and physical self‐care, lifestyle changes during pregnancy, preparing for children including sorting out education, and shared parenting responsibilities, with these episodes ideally led by women sharing personal experiences. There was also a strong emphasis placed on involving men to engage them in fatherhood and support their partners through a dedicated episode on navigating male roles in parenting. Additionally, consumers indicated that while professional advice on evidence‐based health care and guidance on accessing mental health resources is important, it should be balanced to avoid making episodes overly didactic.

Women raised several key discussion points for modifying the Baby Ready Dashboard which focussed largely on accessibility challenges to using the app. Suggestions were made to reduce resource wordiness and increase translation options, particularly for women of lower socio‐economic and literacy status. Auditory resources, along with greater iconography and colourful visuals, were also recommended to enhance engagement amongst women of this demographic. Concerns were also noted about cultural specificity; while topics like alcohol and smoking may resonate with some communities, they could disengage others, highlighting the need for a broader range of issues. Women also outlined that while the app itself should be kept relatively accessible and simple, key contact details for helplines and guidelines should be displayed if mothers wanted to further educate themselves on topics. Proposals were also given on how to launch increase engagement. Suggestions included community organisations, specific social media groups, planning groups, kindergartens, clinics and religious groups. Promoting at university and social societies and to organisations registered to provide sexual educations to schools in a child‐friendly manner to intervene at a young age could be considered. This information may be important to people studying at university as some people may be exploring options related to pre‐conception and pregnancy.

## 4 Discussion

This is the first paper investigating the experiences of women of reproductive age from priority populations and their needs regarding pre‐conception health from a digital resource. Three major themes that emerged from the analysis indicate the importance of culturally specific practices, the need to share knowledge across women and their families, and the benefits of being able to access information in their language.

The complexity of culturally specific practices with both positive and negative influences was discussed. Culturally specific practices described included force, false beliefs and family pressures. These influences were perceived to negatively impact women's wellbeing. There is notable evidence for the influence of the parental home (original home of pregnant women), and traditions and rituals on the wellbeing of women [16]. However, we are the first to report perspectives of women that highlight the potentially detrimental effects of family pressure on women's health during pregnancy and when trying to conceive. Alternatively, positive family support, particularly from the male partner, friends and relatives has been associated with positive outcomes [16]. Outcomes include the mother being less likely to have emotional symptoms, greater levels of self‐efficacy in maternal roles and less susceptibility to, less interpersonal violence [17, 18, 19].

There is mixed evidence in the past literature for the impact of digital interventions for preconception and inter‐conception care outcomes for women from diverse backgrounds [20, 21]. ‘Gabby’, a virtual conversational agent, was trialled to assess its impact on preconception risks experienced by 528 African American and Black women. The use of Gabby resulted in a 16% increase in the reported rate of preconception care risk behaviours being actioned or maintained compared with a control group receiving a letter listing their risks and suggesting they discuss them with a clinician [21]. Additionally, a recent randomised control trial tested a smartphone application intervention to promote healthy weight gain during pregnancy among other dietary factors including healthy diet and physical activity. While differences in gestational weight gain were not statistically significant, women in the intervention group who were obese or overweight prior to pregnancy gained a lesser number of kilograms compared to women in the control group [22]. While mobile technology or digital platforms may not necessarily always show improvements in physiological outcomes such as weight or reduced incidence of illness, there is evidence these digital platforms show improvement in behaviour change, which is crucial to improving and maintaining maternal health [20].

The suggested culturally responsive changes documented in this paper are currently being integrated into the BabyReady? dashboard as follows: We have recorded podcasts with women from multicultural backgrounds at various stages of their reproductive health journey and pregnancy intentions in response to consumers reporting that hearing other women's stories around dealing with cultural pressures would be beneficial. We are also working on translating content, linking in with Chinese social media platforms, and mapping culturally responsive health services.

Limitations to this study included our small sample size, lack of linguistic diversity and generalisability of our findings to other cultural demographics. Additionally, all participants were tertiary educated which means there may have been participant bias in responses around from which resources preconception health information was sought. However, a strength to our study is that we were able to involve consumers from a diverse range of ethnic backgrounds and linguistic diversity.

## 5 Conclusion

Women from East Asian, South Asian, Central Asian, Middle‐Eastern, and African backgrounds may benefit from a tailored digital preconception health dashboard if it includes information on topics such as stress management, how to balance cultural expectations and beliefs with health advice, how to locate female health practitioners who speak their language and appropriate pregnancy planning guidance. Delivering preconception health life experiences through translated resources and podcasts with women from similar backgrounds was also identified as beneficial in engaging these priority groups.

## Author Contributions

**Asvini K. Subasinghe:** conceptualisation, funding acquisition, formal analysis, methodology, writing – original draft, project administration. **Sanduni Madawala:** formal analysis, methodology, project administration, writing – review and editing. **Dinali Fernando:** methodology, project administration, writing – review and editing. **Jue Xie:** writing – review and editing, resources, conceptualisation. **Jessica Watterson:** conceptualisation, writing – review and editing, resources. **Ling Wu:** conceptualisation, writing – review and editing, resources. **Chris Prawira:** resources, methodology. **Patrick Olivier:** conceptualisation, writing – review and editing, resources. **Jacqueline A. Boyle:** conceptualisation, writing – review and editing.

## Ethics Statement

Ethics approval was sought from the Monash University Human and Research Ethics Committee (Project ID: 40436).

## Consent

Participants were provided with a participant information sheet and consent was implicit upon attending workshops.

## Conflicts of Interest

The authors declare no conflicts of interest.

## Data Availability

Data can be made available through reasonable request to the corresponding author.
